# A Systematic Review and Meta-Analysis of Conventional Versus Robotic-Assisted Total Knee Arthroplasty

**DOI:** 10.7759/cureus.46845

**Published:** 2023-10-11

**Authors:** Zien Alabdin Fozo, Ahmed Hussein Ghazal, Mohamed Hesham Gamal, Sajeda Ghassan Matar, Ibrahim Kamal, Khaled Mohamed Ragab

**Affiliations:** 1 Orthopaedics, Ysbyty Gwynedd Hospital, Bangor, GBR; 2 Orthopaedics, Northwick Park Hospital, London North West University Healthcare NHS Trust, Harrow, GBR; 3 Pharmacology and Therapeutics, Faculty of Pharmacy, Tanta University, Tanta, EGY; 4 Pharmacy, Applied Science Private University, Amman, JOR; 5 General Medicine, Al-Azhar University, Alexandria, EGY; 6 Faculty of Medicine, Minia University, Minia, EGY

**Keywords:** systematic review and meta analysis, conventional therapy, arthroplasty, total knee replacement (tkr), total knee replacement, robot‐assisted

## Abstract

This study aims to compare the outcomes and advantages of total knee arthroplasty (TKA) performed using conventional surgical techniques with those conducted using robotic-assisted methods in terms of operation time, Oxford knee score, range of motion, tourniquet time, and Western Ontario and McMaster Universities Arthritis index. We performed a literature search through five databases, namely, PubMed, Cochrane Central, Scopus, Embase, and Web of Science, from inception until July 3, 2023. Randomized clinical trials (RCTs) and cohorts comparing conventional TKA with robotic-assisted TKA were included. The risk of bias of the included RCTs was determined using the Cochrane risk of bias tool and the National Institutes of Health tool for cohort studies. We conducted a meta-analysis using Review Manager 5.4. To analyze continuous data, we calculated the mean difference (MD) along with its corresponding 95% confidence interval (CI). By synthesizing data from a comprehensive analysis, the study unveiled noteworthy distinctions between robotic-assisted arthroplasty and conventional arthroplasty across critical parameters. First, a substantial alteration in the hip-knee-ankle (HKA) angle was observed, with the robotic-assisted approach demonstrating a significant difference (MD = 0.84, 95% CI = 0.25-1.43, p = 0.005). Second, in terms of operative time, a notable reduction in surgical duration was noted with conventional TKA (MD = 16.85, 95% CI = 8.08-25.63, p = 0.0002). The assessment of tourniquet time exhibited a significantly longer duration for robotic-assisted arthroplasty (MD = 35.70, 95% CI = 27.80-43.61, p < 0.001). Our findings indicate that conventional TKA outperforms robotic-assisted TKA, primarily due to its shorter operative and tourniquet times, along with a more favorable change in the HKA angle. However, it is worth noting that robotic-assisted TKA showed a slight advantage in pain outcomes, although this advantage was not statistically significant. To gain a more comprehensive understanding, we recommend conducting a large-scale randomized controlled trial that directly compares both TKA methods. This trial should evaluate costs and long-term outcomes while ensuring consistent follow-up durations among studies. Such an approach would greatly assist orthopedic decision-making and contribute to improved TKA outcomes.

## Introduction and background

Osteoarthritis is a common disability worldwide [1]. Primary osteoarthritis is considered a degenerative condition, meaning that it results from the wear and tear of the joint over time. It is considered the most common joint disease or reason for disability in the United States [2]. Among individuals aged 60 and above, knee osteoarthritis affects 13% of women and 10% of men [3]. The increased rate in females may be related to pregnancy as it decreases calcium levels in the blood and bones [4]. Knee osteoarthritis is a degenerative disease and develops progressively over a decade [5]. Knee osteoarthritis can be classified into primary, which develops without a clear reason, and secondary, which develops due to a clear reason such as increased force on the joint, obesity, joint injuries, bone deformities, or certain metabolic diseases [3,4].

Total knee arthroplasty (TKA) is considered one of the most common procedures. In 2014, about 700,000 TKA procedures were performed in the United States [6]. This procedure enhances the quality of life of patients and decreases pain associated with osteoarthritis [7]. Although more than 20% of patients remain unsatisfied with the outcomes of this procedure, it is considered a cost-effective procedure that enhances the quality of life of a patient by saving about 1,000-12,000 euros for the patient [8].

TKA has traditionally been considered a therapeutic option for advanced stages of knee osteoarthritis [9]. In the conventional approach, surgeons rely on their skills and experience for precise bone cuts and implant positioning. While they use guides and instruments, there is a potential for slight variations. Therefore, robotic-assisted approaches have been implemented to increase precision [10]. On the other hand, the robotic-assisted approach is associated with increased operative time and cost as it requires more expensive equipment [10]. There are three types of robotic systems, namely, autonomous, hands-on, and passive, which differ in terms of surgeon control of the operation [11]. Robotics can assist in performing a minimally invasive approach more consistently, potentially reducing tissue damage and improving recovery times. In our study, we aim to compare variable outcomes related to efficacy between conventional and robotic-assisted TKA.

## Review

Methodology

This systematic review and meta-analysis was conducted and reported in accordance with both the Cochrane guidelines and the Preferred Reporting Items for Systematic Reviews and Meta-Analyses (PRISMA) guidelines [12,13].

Search Strategy

A systematic search was conducted through PubMed, Web of Science, Cochrane Library, and Scopus until July 2023 using the following search strategy: (robot OR robotic OR “robotic surgical procedure” OR “robotic arm assisted”) AND (Arthroplasty OR Replacement) AND (Knee OR Knees). Two authors independently reviewed the literature for articles that matched the inclusion and exclusion criteria using the search strategy, while both authors independently extracted the data from the included articles.

Study Selection and Eligibility Criteria

All included articles were in the English language. The included articles were manually screened to guarantee that all matched articles were included. We included studies that involved patients who underwent TKA whether by robotic surgery, as our main intervention, or in a conventional way by surgeons. We included randomized controlled trials (RCTs) and observational studies. We excluded case reports, case series, reviews, letters, or studies involving unicompartmental, not total knee, arthroplasty. We measured the following outcomes: hip-knee-ankle (HKA) angle (change), hospital for special surgery (HSS), operation time, Oxford knee score, range of motion (ROM), tourniquet time, and Western Ontario and McMaster Universities Arthritis (WOMAC) index.

Quality Assessment

The Cochrane risk of bias tool (version 1) was used to evaluate the included RCTs [14]. This tool comprises the following categories: (1) identification of selection bias and other potential biases; (2) allocation of study groups; (3) blinding of participants and investigators; (4) evaluation of outcomes and the use of blinding in this assessment; and (5) randomization of the study population. The possibility of bias in judgment can be a high, low, or unclear risk of bias.

Data Extraction

Using Excel sheets, the extracted data contained the following items: (1) summary including study ID, site, study design, inclusion criteria, main outcomes/endpoint, and conclusion; (2) baseline data including study arms, sample size, age, follow-up, gender, operation side, underlying diagnosis, ROM, and preoperative knee score. Further, the following outcomes were used: (a) change in HKA angle, (b) HSS, (c) operative time, (d) Oxford knee score, (e) ROM, (f) tourniquet time, (g) WOMAC pain score.

Data Synthesis

We utilized RevMan version 5.4 for the statistical analysis in this study. We set the significance level at <0.05. For continuous data, we computed the mean difference (MD) and determined the 95% confidence interval (95% CI). Additionally, we assessed heterogeneity using both the I^2^ and the chi-square test. Data were considered heterogeneous if the chi-square test yielded a p-value <0.1 and if the I^2^ value exceeded 50%. Homogeneous data were analyzed using a fixed-effect model, while a random-effects model was applied for heterogeneous data.

Results

Literature Search

**Figure 1:**
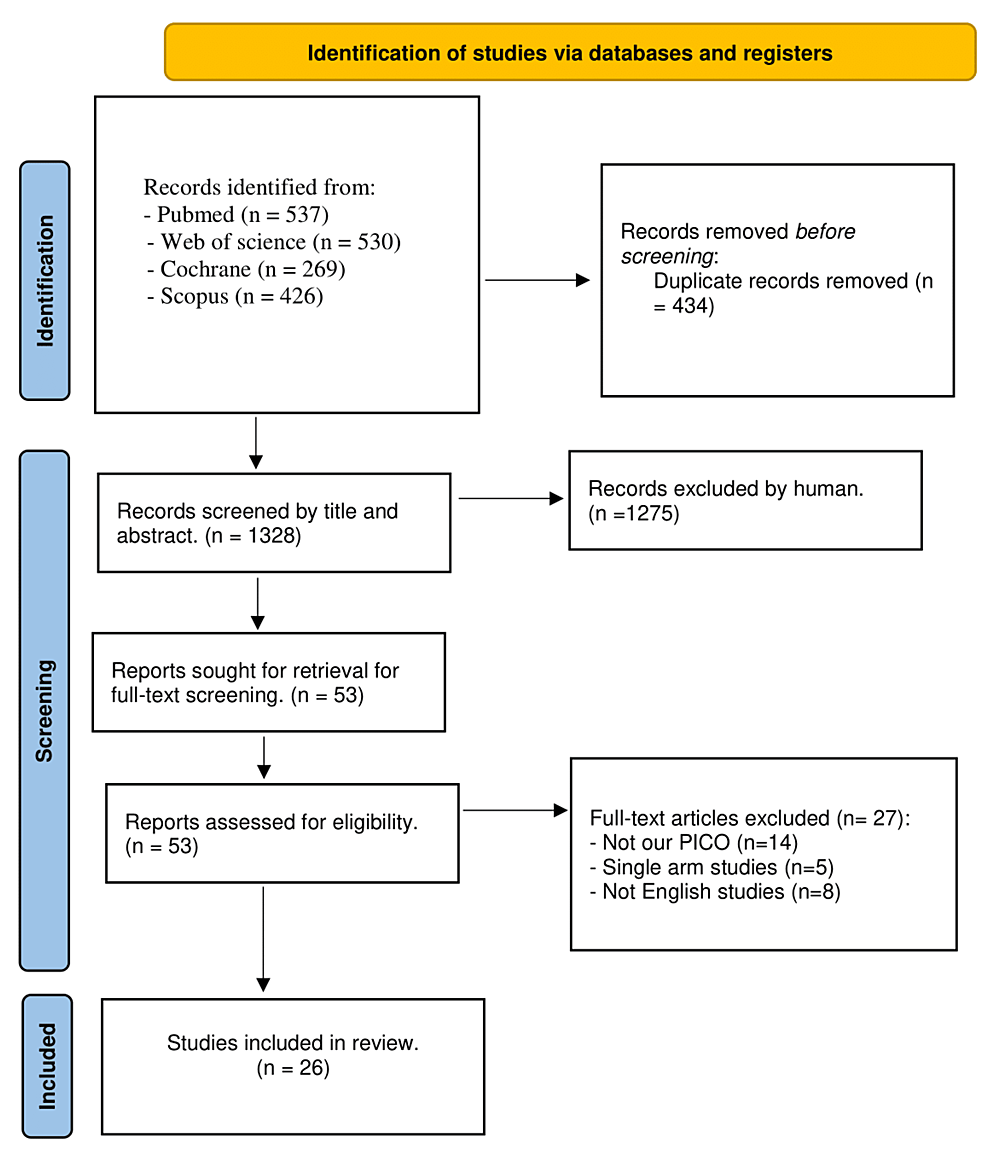
Preferred Reporting Items for Systematic Reviews and Meta-Analyses flow diagram.

We identified a total number of 1,762 results related to the topic. After removing duplicates, 1328 results remained. After manual screening, 53 articles were included for full content screening. After full content screening, 26 studies were included in the final analysis [15-40] (Figure 1).

Characteristics of the Included Studies and Patients

Our study encompassed a diverse range of research methodologies, comprising nine RCTs, one case-control study, three prospective cohorts, and 13 retrospective cohort studies. In total, our analysis involved 9,964 patients drawn from various countries, including the United States, China, South Korea, Belgium, the United Kingdom, Greece, Singapore, Germany, and Thailand. The study participants were predominantly elderly, aged over 60 years, with a higher proportion of females undergoing TKA compared to males. Furthermore, the primary indication for TKA across the majority of patients was osteoarthritis, albeit in varying stages (Table 1).

**Table 1:** Summary and baseline characteristics of the included studies. TKA = total knee arthroplasty; ROM = range of motion

Study ID	TKA techniques, n (%)	Study design	Site	Follow-up, years	Age, (mean ± SD), years	Male, n (%)	Operation side, n (%)	Underlying diagnosis, n (%)	Preoperative knee score, (mean ± SD)	ROM, (mean ± SD)	Inclusion criteria	Main outcomes	Conclusions
Bolam et al. 2022 [15]	Robotic-assisted TKA (RA TKA), 53 (38.97)	Prospective cohort study	USA	1.775 (SD = 0.75)	70.3 ± 8.6	19 (36)	1. Right, 21 (40) 2. Left, 32 (60)	Osteoarthritis	NR	NR	1. Between February 2020 and November 2021. 2. Performed at least 10 RA TKA procedures. 3. Consecutive patients undergoing primary TKA for osteoarthritis. 4. Aged 70.3 (SD 8.6) years	1. Cumulative summation (CUSUM) analysis of the initial RA total knee arthroplasty. 2. Operative complications	“The introduction of the RA TKA system was associated with a learning curve for operative time of 8.7 cases. Operative times between the RA TKA and conventional TKA groups were similar. The short learning curve implies this RA TKA system can be adopted relatively quickly into a surgical team with minimal risks to patients”
	Conventional TKA, 83 (61.03)				70.5 ± 9.1	30(36)	1. Right, 44(53) 2. Left, 39(47)						
Cai et al. 2022 [16]	RA TKA, 40 (48.78)	RCT	China	Mean (3)	66.98 ± 4.84	25 (63)	NR	Osteoarthritis	NR	65.18 ± 9.45	1. Age ≥60 years old. 2. Initial postoperative unilateral TKA surgery because of knee osteoarthritis. 3. Rehabilitation treatment in the rehabilitation unit	1. Hospital for special surgery Knee Rating Score. 2. Modified Barthel Index. 3. Range of motion (degrees)	“The robot-assisted rehabilitation training program is an effective intervention that significantly improves the daily activity ability and knee function of older adults following TKA”
	Conventional TKA, 42 (51.22)				65.55 ± 5.30	28(67)				65.19 ± 9.13			
Cho et7 al. 2018 [17]	RA TKA, 160 (41.03)	Retrospective cohort study	South Korea	10.8 (SD = 0.9)	68.2 ± 3.83	14 (8.75)	1. Right, 78 (48.75). 2. Left, 82 (51.25)	Osteoarthritis	20.3 ± 9.1	124.8 ± 4.6	1. Underwent primary total knee arthroplasty. 2. Using a specific total knee system. 3. With a minimum follow-up of ten years. 4. Aged 68.2 (57–80) years	1. KSS score. 2. Range of motion (degrees). 3. WOMAC score. 4. SF-12 physical score	“Our study showed excellent survival with both robotic and conventional TKA and similar clinical outcomes at long-term follow-up. And, in terms of radiological outcome, robotic TKA showed better accuracy and consistency with fewer outliers compared with conventional TKA. With a longer follow-up and larger cohort, the accuracy and effectiveness of robotic TKA on implant survival rate can be elucidated in the future”
	Conventional TKA, 230 (58.97)			11.2 (SD = 1.1)	67.6 ± 4.167	33 (14.35)	1. Right, 110 (47.83). 2. Left, 120 (52.17)		23 ± 10	119.8 ± 10.2			
De Grave et al. 2022 [37]	Robotic inverse alignment, 40 (33.33)	Retrospective cohort study	Belgium	Mean (1)	69.7 ± 9.1	15 (37)	NR	Knee osteoarthritis	26.3 ± 6.4	NR	1. Patients receiving TKA after end-stage knee osteoarthritis. 2. All patients provided informed consent. 3. At least one-year follow-up. 4. Aged 69.1 (SD = 9.5) years	1. Oxford knee score 2. Operative complications	“The results of this study suggest that the introduction of both patient-specific alignment and robotically assisted surgery improve clinical outcomes in TKA surgery. When access to robotic assistance is available, performing patient-specific alignment should be the objective”
	Conventional mechanical alignment, 40 (33.33)				66.8 ± 9.7	17 (62)			27.2 ± 5.2				
	Robotic mechanical alignment, 40 (33.34)				69.1 ± 9.5	12 (30)			23.7 ± 7.9				
He et al 2022 (1), [18]	RA TKA, 30 (33.33)	Retrospective cohort study	China	At least 11 months	71.3 ± 7.2	7 (23.33)	NR	Osteoarthritis	45.3 ± 8.4	NR	1. Patients with only advanced knee OA. 2. The OA KellgreneLawrence classiﬁcation was grade IV. 3. Varus deformity of no more than 15 4. Without extra-articular deformity	1. KSS score. 2. HKA axis score	“Compared to the PSI and CO, RA is more minimally invasive and more accurate in radiographic results”
	Patient-speciﬁc instrumentation TKA, 30 (33.33)				68.7 ± 9.7	8 (26.67)			49.6 ± 10.2				
	Conventional TKA, 30 (33.34)				66.8 ± 6.5	8 (26.67)			47.6 ± 9.4				
He et al. 2022 (2), [19]	RA TKA, 30 (50)	Retrospective cohort study	China	Mean (1)	71.3 ± 7.2	7 (23.33)	1. Center, 18 (60) 2. Right, 12 (40)	Osteoarthritis	NR	Range: 10.5–108.7	1. Age ≤80 years. 2. Patients with only deformity of the knee. 3. Varus deformity of no more than 15. 4. Without extra-articular deformity	1. Posterior condylar angle change. 2. Range of motion (degrees)	“The accuracy of femoral rotational alignment reconstructed achieved by RATKA is signiﬁcantly better than that of COTKA and is more conducive to the recovery of knee ﬂexion function after surgery; although RATKA reduces intraoperative blood loss and postoperative LOS, the short-term clinical efﬁcacy comparison has not yet demonstrated the advantages of robotic technology, and a more optimized design is needed to improve the efﬁciency of RATKA surgery”
	Conventional TKA, 30 (50)				66.8 ± 6.5	8(26.67)	1. Center, 17 (56.67) 2. Right, 13 (43.44)			Range: 7.3–110.4			
Jeon et al. 2019 [20]	RA TKA, 84 (51.53)	Retrospective cohort study	South Korea	10.725 (9–11.65)	69.2 ± 6.167	18 (23)	1. Right, 35 (41.7) 2. Left, 49 (58.3)	Primary osteoarthritis	41.7 ± 16.1	117 ± 7.3	1. Patients who underwent TKA. 2. Between October 2006 and October 2009. 3. Written informed consent was obtained from all patients. 4. Aged 69.5 (47–83) years	1. Hip-knee-ankle angle. 2. Range of motion (degrees). 3. KSS score	“Robot-assisted TKA does not improve long-term clinical or radiologic outcomes compared with conventional TKA”
	Conventional TKA, 79 (48,47)			10.79(9.01–12.3)	70.1 ± 40.5	10 (18.5)	1. Right, 38(48.1) 2. Left, 41(51.9)		45.1 ± 18.1	116.7 ± 8.2			
Kayani et al. 2018 [21]	RA TKA, 60 (50)	Prospective cohort study	UK	NR	67.6 ± 7.6	28 (46.7)	1. Right, 33 (55) 2. Left, 27 (45)	Symptomatic osteoarthritis	NR	NR	1. Patients with symptomatic knee osteoarthritis undergoing primary TKA. 2. Between 2016 and 2017. 3. Patients between 18 and 80 years of age	1. Surgical team anxiety levels. 2. Operative time. 3. operative complications	“Implementation of robotic-arm assisted TKA led to increased operative times and heightened levels of anxiety amongst the surgical team for the initial seven cases but there was no learning curve for achieving the planned implant positioning. Robotic-arm-assisted TKA improved the accuracy of implant positioning and limb alignment compared to conventional jig-based TKA. The findings of this study will enable clinicians and healthcare professionals to better understand the impact of implementing robotic TKA on the surgical workflow, assist in the safe integration of this procedure into surgical practice, and facilitate theatre planning and scheduling of operative cases during the learning phase”
	Conventional TKA, 60 (50)				68.7 ± 6.1	27 (45)	1. Right, 29 (48.3) 2. Left, 31 (51.7)						
Kenanidis et al. 2022 [22]	RA TKA, 30 (50)	Prospective cohort study	Greece	Mean(half year)	69.3 ± 6.8	6 (20)	1. Right, 18(60) 2. Left, 12(40)	Symptomatic primary unilateral end-stage knee OA	13.9 ± 4.7	NR	1. Consecutive primary unilateral raTKAs with ROSA knee system. 2. Between September 2020 and May 2021. 3. Adult patients suffering from symptomatic primary unilateral end-stage knee OA	1. Length of hospital stay. 2. VAS score. 3. OKS score. 4. The Forgotten Joint Score	“raTKA was associated with the same complication risk, less pain level, better patient satisfaction, and PROMs on 6-month follow-up than the mTKA group”
	Manual TKA, 30 (50)				69.1 ± 7	6 (20)	1. Right, 22(73.33) 2. Left, 8(26.67)		15.8 ± 5.2				
Kim et al. 2019 [23]	RA TKA, 724 (50)	RCT	South Korea	13 (SD = 5)	60 ± 7	132 (19.58)	Both	Osteoarthritis	NR	NR	1. From January 2002 to February 2008. 2. Robotic-assisted TKAs in 850 patients and 990 conventional TKAs. 3. Patients younger than 65 years. 4. Had an end-stage of osteoarthritis of both knees	Radiographic results	“At a minimum follow-up of 10 years, we found no differences between robotic-assisted TKA and conventional TKA in terms of functional outcome scores, aseptic loosening, overall survivorship, and complications. Considering the additional time and expense associated with robotic-assisted TKA, we cannot recommend its widespread use”
	Conventional TKA, 724 (50)				61 ± 8	144 (21.36)							
Lau et al. 2023 [24]	RA TKA, 71 (50)	Retrospective cohort study	Hong Kong	Mean(1)	69.58 ± 7.47	40 (50)	1. Left, 42 (56) 2. Right, 29 (43.3)	End‐stage knee OA	42.40 ± 19.38	97.18 ± 16.6	1. All patients who were age over 40 years old. 2. Undergoing TKA for end‐stage knee OA. 3. Kellgren and Lawrence grade 3–4. 4. After failing a minimum of 8 weeks of non‐operative management	1. ROM score. 2. KSS score. 3. KFS score	“Robotic‐assisted TKA achieved a lower rate of mechanical axis Outlier in the coronal and sagittal plane with a shorter hospital stay. Yet both methods achieve a similar functional outcome”
	Conventional TKA, 71 (50)				68.55 ± 7.87	40 (50)	1. Left, 33 (44) 2. Right, 38 (56.7)		41.10 ± 13.08	98.38 ± 13.62			
Lee et al. 2023 [25]	RA TKA, 194 (22.69)	Retrospective cohort study	South Korea	11.9 (SD = 1.5)	71.8 ± 8.2	18 (9.28)	NR	Primary osteoarthritis of the knee	22.4 ± 7.5	125.1 ± 13.6	1. Between January 2004 and December 2009. 2. Underwent TKA for knee osteoarthritis. 3. Their MA was between 20° varus and 10° valgus. 4. Had a minimum follow-up of 10 years	1. ROM score. 2. KSS score. 3. WOMAC scores	“Our study demonstrated satisfactory survival rates for robotic, navigational, and conventional TKAs and similar clinical outcomes during the long-term follow-up. Larger studies with continuous serial data are needed to conﬁrm these ﬁndings”
	Conventional TKA, 270 (31.58)			11.8 (SD = 1.5)	71 ± 7	20 (7.4)			22.8 ± 10.4	123.2 ± 17.3			
	Navigational, 391 (45.73)			12 (SD = 1.4)	71.6 ± 8.1	26 (6.65)			23.4 ± 9.8	123.1 ± 14.7			
Li et al. 2022 [26]	RA TKA, 73 (48.67)	RCT	China	Mean(0.25)	68 ± 7.56	13 (17.8)	1. Left, 36 (49.3) 2. Right, 37 (50.7)	Primary knee osteoarthritis	NR	NR	1. From January 2020 to March 2021. 2. Underwent TKA for primary knee osteoarthritis. 3. Older than 18 years old	1. ROM score. 2. KSS score. 3. WOMAC scores	“HURWA robotic-assisted TKA is safe and effective, resulting in better alignment for mechanical axis than conventional TKA. The improvement in knee ﬂexion and functional recovery after HURWA robotic-assisted TKA were similar to those after conventional TKA. However, longer follow-up is needed to determine whether the improved alignment of the mechanical axis will produce better long-term clinical outcomes”
	Conventional TKA, 77 (51.33)				69 ± 5.29	15 (19.5)	1. Left, 47 (61) 2. Right, 30(39)						
Liow et al. 2014 [27]	RA TKA, 31 (51.67)	RCT	Singapore	Mean(0.5)	67.5 ± 8.6	NR	NR	Primary knee osteoarthritis	34.2 ± 14.6	121.0 ± 17.4	1. Recruited based on the diagnosis of primary knee osteoarthritis. 2. With genu varus deformity and a ﬁxed ﬂexion deformity of less than 15°. 3. From May 2012 to December 2012	1. ROM in degrees. 2. OKS score. 3. SF-36 score	“Robot-assisted TKA produces similar short-term clinical outcomes when compared to conventional methods with reduction of MA alignment and joint-line deviation outliers”
	Conventional TKA, 29 (48.33)				68.3 ± 7.7				34 ± 17.1	119.8 ± 17.9			
Marchand et al. 2019 [28]	RA TKA, 53 (50)	Retrospective cohort study	USA	At least one	65 ± 7	25 (47)	NR	Primary knee osteoarthritis	NR	NR	1. Between September 16, 2016, and March 16, 2017. 2. A total of 153 RAA TKAs were performed. 3. Due to Primary knee Osteoarthritis. 4. Had a minimum follow-up of One year	1. WOMAC scores. 2. Physical function scores	“The RAA technique was found to have the strongest association with improved scores when compared with age, gender, and BMI. This study suggests that RAA patients may have short-term improvements at a minimum of 1 year postoperatively. However, longer-term follow-up with greater sample sizes is needed to further validate these results”
	Manual TKA, 53 (50)				63 ± 8	28 (53)							
Marchand et al. 2020 [29]	RA TKA, 140 (70)	Retrospective cohort study	USA	At least one	65 ± 9	25 (42)	NR	Primary knee osteoarthritis	NR	NR	1. RATKA and 60 manual TKA cases. 2. From April 20, 2016, to June 24, 2016. 3. Due to primary knee osteoarthritis. 4. Had a minimum follow-up of one year	Mean operative time (minutes)	“The results of this study are important because they demonstrate how the complexity of a technology which initially increases operative time can be overcome and become more time-effective than conventional techniques”
	Manual TKA, 60 (30)				63 ± 11	27 (45)							
Nam et al. 2022 [30]	RA TKA, 154 (50)	Retrospective cohort study	South Korea	Mean (0.5)	70.8 ± 6.1	20 (18.18)	NR	1. Osteoarthritis, 152 (98.7) 2. Osteonecrosis, 2 (1.3)	NR	NR	1. Between July 2020 and December 2020. 2. A consecutive series of 162 primary TKAs. 3. Due to primary knee osteoarthritis and osteonecrosis. 4. Preoperative Kellgren-Lawrence grade IV varus knee	1. Radiologic results. 2. Hip knee angle axis	“Robot‑assisted TKA showed improved mechanical axis and higher accuracy of component positioning compared to the conventional TKA technique, with no significant difference in polyethylene liner thickness between the two groups. Long‑term follow‑up studies are needed to compare the clinical outcomes of robot‑assisted TKA”
	Conventional TKA, 154 (50)				70.7 ± 6.3	20 (18.18)		1. Osteoarthritis, 152 (98.7) 2. Osteonecrosis, 2 (1.3)					
Naziri et al. 2019 [31]	RA TKA, 40 (50)	Retrospective cohort study	USA	Mean (0.25)	Mean (69.5)	24 (60)	1. Right, 23 (57.5) 2. Left, 17 (42.5)	NR	Mean (81.5)	Mean (117.5)	1. Adults (> 18 years of age). 2. Required primary TKA and were willing and able to comply with postoperative follow-up. 3. Comply with postoperative follow-up appointment requirements and self-evaluations	1. Range of motion 2. KSS score. 3. Rate of complications	“Despite comparable outcomes, the learning curve for raTKA appeared to progress rapidly”
	Traditional TKA, 40 (50)				Mean (70.9)	24 (60)	1. Right, 21 (52.5) 2. Left, 19 (47.5)		Mean(77.3)	Mean(118.5)			
Savov et al. 2021 [32]	RA TKA, 70 (50)	Case control study	Germany	NR	Mean (64.4)	22 (31.43)	NR	Osteoarthritis either primary or post-traumatic	NR	NR	1. Between March 2018 and March 2020. 2. Consecutive patients who underwent primary TKA. 3. Received robotic-assisted TKA or conventional TKA. 4. Primary osteoarthritis or post-traumatic	1. HKA angle axis. 2. Medial proximal tibia angle	“After completing the initial learning curve of 11 cases, the surgery time required to perform imageless robotic handpiece-assisted TKA was similar to that for the conventional technique. However, no learning curve was observed for the implant positioning when using the imageless robotic system. The implementation of the intraoperative plan was accurate up to < 2°. The precision of the system allows the implementation of different joint balancing approaches between valgus and varus morphotypes”
	Conventional TKA, 70 (50)				Mean (65.9)	20 (28.57)							
Song et al. 2011 [33]	RA TKA	RCT	South Korea	1.33 (SD = 0.267)	67 ± 6.3	0	NR	Primary osteoarthritis of the knee	NR	120 ± 16	1. Primary osteoarthritis of the knee. 2. No previous hemiarthroplasty or TKA. 3. A mechanical axis between 20 varus and 5 valgus. 4. No severe instability that could not be treated by cruciate-retaining TKA	1. Radiologic results. 2. Range of motion in degrees	“The better alignment accuracy of robotic TKA and the good clinical results achieved may favorably inﬂuence clinical and radiological outcomes”
	Conventional TKA									123 ± 14.3			
Song et al. 2012 [34]	RA TKA, 50 (50)	RCT	South Korea	At least Three	66.1 ± 7.1	4 (8)	NR	Patients with primary osteoarthritis of the knee	NR	125 ± 7.6	1. Between July 2004 and September 2005. 2. Patients with primary osteoarthritis of the knee. 3. A mechanical axis between 20 degrees and 5 degrees valgus. 4. No severe instability that could not be treated by cruciate-retaining TKA	1. ROM in degrees. 2. WOMAC scores	“Robotic-assisted TKA appears to reduce the number of mechanical axis alignment outliers and improve the ability to achieve ﬂexion-extension gap balance, without any differences in clinical scores or complications when compared to conventional manual techniques”
	Conventional TKA, 50 (50)				64.8 ± 5.3	5 (10)				123 ± 12.3			
Thiengwittayaporn et al. 2021 [35]	RA TKA, 75 (49.34)	RCT	Thailand	Mean (0.75)	69 ± 8.3	6 (8)	1. Right, 40 (53.33) 2. Left, 35 (46.67)	Primary knee osteoarthritis	27.8 ± 5.5	122 ± 14.1	1. Between March 2020 and January 2021. 2. Patients with primary knee osteoarthritis. 3. Could not be treated with conservative measures. 4. Ages between 40 and 80 years were included for a primary TKA	1. Hip knee Ankle axis. 2. An overall mechanical alignment. 3. Postoperative radiographic outcomes	“The imageless RATKA has better alignment accuracy with a short learning curve; thus, it presents an attractive option for TKA”
	Conventional TKA, 77 (50.66)				69.1 ± 7.3	15 (19.48)	1. Right, 45 (58.44) 2. Left, 32 (41.56)		27.1 ± 5.7	126 ± 14.1			
Tompkins et al. 2021 [36]	RA TKA, 2,392 (50)	Retrospective cohort study	USA	Mean (0.25)	68.6 ± 8.7	1,027 (43)	NR	Primary osteoarthritis	NR	NR	1. Between January 1, 2017 and December 31, 2019. 2. By 6 high volume surgeons in each cohort. 3. Undergone TKA with primary osteoarthritis. 4. Aged 68.6 (SD = 8.7)y	1. Operative complications. 2. clinical readmissions	“RTKA was a longer and costlier procedure than MTKA for experienced surgeons, without clinically significant differences in LOS or complications. Home health care was utilized more often after RTKA, but fewer readmissions occurred after RTKA. Longer term follow up and functional outcome studies are required to determine if the greater cost of RTKA is offset by lower revision rates and/or improved functional results”
	Manual TKA, 2,392 (50)				68.6 ± 8.5	1,031 (43)							
Xu et al. 2021 [38]	RA TKA, 37 (51.39)	RCT	China	Mean (0.25)	64.5 ± 5.3	11 (29.7)	1. Left, 21 (56.8) 2. Right, 16 (43.2)	End-stage KOA unresponsive to conservative treatment	32 ± 8	105 ± 7.5	1. From June 2020 to December 2020. 2. Patients scheduled to undergo initial unilateral TKA. 3. Patients included understanding of the beneﬁts and risks of this trial. 4. Diagnosis of end-stage KOA unresponsive to conservative treatment. 5. Age 18–80 years, with no gender restriction	1. ROM in degrees. 2. KSS score 3. WOMAC scores	“RA-TKA requires more time than CM-TKA, which may be related to the learning curve and intraoperative registration. The short-term postoperative knee functional outcomes had no differences between the two groups, and RA-TKA improved the accuracy of tibial component alignment. Further follow-up studies are required to investigate the long-term outcomes”
	Conventional TKA, 35 (48.61)				63.4 ± 7.2	7 (20)	1. Left, 16 (45.7) 2. Right, 19 (54.3)		28 ± 4.75	105 ± 7.5			
Xu et al. 2022 [39]	RA TKA, 17 (51.51)	RCT	China	Mean (0.5)	66.6 ± 3.7	3 (17.65)	NR	End-stage KOA unresponsive to conservative treatment	109.3 ± 25.8	NR	1. Age between 18 and 80 years. 2. A diagnosis of end-stage osteoarthritis, Kellgren–Lawrence (KL) staging III-IV. 3. No intraarticular puncture and drug injection and no periarticular drug application in the last three months. 4. Diagnosis of end-stage KOA unresponsive to conservative treatment. 5. Age 18-80 years, with no gender restriction	1. Inflammatory markers after surgery. 2. Radiologic results	“Compared with CM-TKA, RA-TKA decreases rather than increases trauma. It might shorten the time required for bone cutting and gap balancing, reduce mechanical errors related to the osteotomy and prosthesis position, and improve the accuracy of the mechanical alignment”
	Conventional TKA, 16 (48.48)				67.3 ± 3.5	3 (18.75)			101.1 ± 32.2				
Yang et al. 2017 [40]	RA TKA, 71 (60.17)	Retrospective cohort study	South Korea	Mean (10.5)	66.3 ± 7.5	3 (4.22)	NR	End-stage KOA unresponsive to conservative treatment	NR	121.7 ± 16	1. From January 2004 to December 2007. 2. Underwent TAK under the diagnosis of knee osteoarthritis. 3. Mechanical axis between 20° varus and 10° valgus. 4. Diagnosis of end-stage KOA unresponsive to conservative treatment. 5. Age 18–80 years, with no gender restriction	1. VAS scores. 2. WOMAC scores. 3. KSS scores. 4. ROM in degrees	“Both robotic and conventional TKAs resulted in good clinical outcomes and postoperative leg alignments. Robotic TKA appeared to reduce the incidence of leg alignment outliers and radiolucent lines compared to conventional TKA”
	Conventional TKA, 42 (39.83)				67.8 ± 6.5	5 (11.9)				122 ± 14.3			

Quality Assessment Results

**Figure 2:**
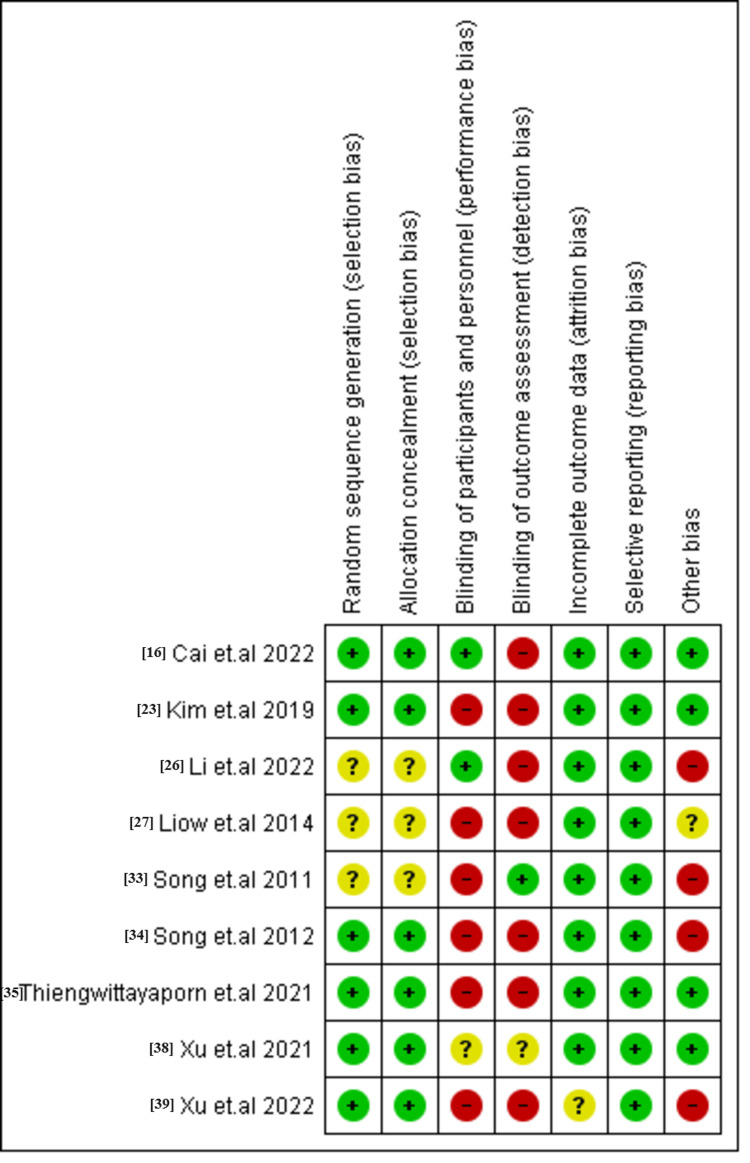
Risk of bias graph summary for randomized controlled trials.

Of the nine included RCTs, seven had a high bias and two were high quality with low bias, as shown in Figure 2. For cohort studies, all studies were fair in quality, and only three studies had good quality. The case-control study exhibited fair quality (Tables 2, 3).

**Table 2:** NIH quality assessment tool for observational cohort and cross-sectional studies.

Study ID	NIH quality assessment tool for observational cohort and cross-sectional studies															Quality rating: good (11–14), fair (7.5–10.5), or poor (0–7), Yes = 1/No = 0.5/NR & NA & CD = 0
	1. Was the research question or objective in this paper clearly stated?	2. Were eligibility/selection criteria for the study population prespecified and clearly described?	3. Were the participants in the study representative of those who would be eligible for the test/service/intervention in the general or clinical population of interest?	4. Were all eligible participants that met the prespecified entry criteria enrolled?	5. Was the sample size sufficiently large to provide confidence in the findings?	6. For the analyses in this paper, were the exposure(s) of interest measured prior to the outcome(s) being measured?	7. Was the time frame sufficient so that one could reasonably expect to see an association between exposure and outcome if it existed?	8. For exposures that can vary in amount or level, did the study examine different levels of the exposure as related to the outcome (e.g., categories of exposure, or exposure measured as a continuous variable)?	9. Were the exposure measures (independent variables) clearly defined, valid, reliable, and implemented consistently across all study participants?	10. Was the exposure(s) assessed more than once over time?	11. Were the outcome measures prespecified, clearly defined, valid, reliable, and assessed consistently across all study participants?	12. Were the people assessing the outcomes blinded to the participants' exposures/ interventions?	13. Was the loss to follow-up after baseline 20% or less? Were those lost to follow-up accounted for in the analysis?	14. Were key potential confounding variables measured and adjusted statistically for their impact on the relationship between exposure(s) and outcome(s)?	Total scores	
	Yes/No/Not reported (NR) or cannot determine (CD) or not applicable (NA)	Yes/No/Not reported (NR) or cannot determine (CD) or not applicable (NA)	Yes/No/Not reported (NR) or cannot determine (CD) or not applicable (NA)	Yes/No/Not reported (NR) or cannot determine (CD) or not applicable (NA)	Yes/No/Not reported (NR) or cannot determine (CD) or not applicable (NA)	Yes/No/Not reported (NR) or cannot determine (CD) or not applicable (NA)	Yes/No/Not reported (NR) or cannot determine (CD) or not applicable (NA)	Yes/No/Not reported (NR) or cannot determine (CD) or not applicable (NA)	Yes/No/Not reported (NR) or cannot determine (CD) or not applicable (NA)	Yes/No/Not reported (NR) or cannot determine (CD) or not applicable (NA)	Yes/No/Not reported (NR) or cannot determine (CD) or not applicable (NA)	Yes/No/Not reported (NR) or cannot determine (CD) or not applicable (NA)	Yes/No/Not reported (NR) or cannot determine (CD) or not applicable (NA)	Yes/No/Not reported (NR) or cannot determine (CD) or not applicable (NA)		
Bolam et al. 2022 [15]	Yes	Yes	Yes	NR	Yes	Yes	Yes	NA	Yes	NR	Yes	NR	Yes	Yes	10	Fair
Cho et al. 2018 [17]	Yes	Yes	Yes	NR	Yes	Yes	Yes	NA	Yes	NR	Yes	NR	Yes	Yes	10	Fair
De Grave et al. 2022 [37]	Yes	Yes	Yes	Yes	NR	Yes	Yes	NA	Yes	NR	Yes	NR	Yes	Yes	10	Fair
He et al. 2022 (1), [18]	Yes	Yes	Yes	Yes	NR	Yes	No	NA	NR	NR	Yes	NR	Yes	Yes	8.5	Fair
He et al. 2022 (2), [19]	Yes	Yes	Yes	Yes	Yes	Yes	Yes	NA	Yes	NR	Yes	NR	Yes	Yes	11	Good
Jeon et al. 2019 [20]	Yes	Yes	Yes	Yes	Yes	Yes	Yes	NA	Yes	NR	Yes	NR	Yes	Yes	11	Good
Kayani et al. 2018	Yes	Yes	Yes	Yes	Yes	Yes	No	NA	NR	NR	Yes	Yes	Yes	Yes	10.5	Fair
Kenanidis et al. 2022 [22]	Yes	Yes	Yes	Yes	NR	Yes	Yes	NA	Yes	NR	Yes	NR	Yes	Yes	10	Fair
Lau et al. 2023 [24]	Yes	Yes	Yes	Yes	NR	Yes	Yes	NA	Yes	NR	Yes	NR	Yes	Yes	10	Fair
Lee et al. 2023 [25]	Yes	Yes	Yes	Yes	Yes	Yes	Yes	NA	Yes	NR	Yes	NR	Yes	Yes	11	Good
Marchand et al. 2019 [28]	Yes	Yes	Yes	Yes	NR	Yes	Yes	NA	Yes	NR	Yes	NR	Yes	Yes	10	Fair
Marchand et al. 2020 [29]	Yes	Yes	Yes	NR	NR	Yes	Yes	NA	Yes	NR	Yes	NR	Yes	Yes	9	Fair
Nam et al. 2022 [30]	Yes	Yes	Yes	Yes	NR	Yes	No	NA	Yes	NR	Yes	NR	Yes	Yes	9.5	Fair
Naziri et al. 2019 [31]	Yes	Yes	Yes	Yes	NR	Yes	No	NA	NR	NR	Yes	NR	Yes	Yes	8.5	Fair
Tompkins et al. 2021 [36]	Yes	Yes	Yes	Yes	Yes	Yes	Yes	NA	NR	NR	NR	NR	Yes	Yes	9	Fair
Yang et al. 2017 [40]	Yes	Yes	Yes	NR	NR	Yes	Yes	NA	Yes	NR	Yes	NR	Yes	Yes	9	Fair

**Table 3:** NIH quality assessment tool for observational case-control studies.

ID	NIH quality assessment tool for observational case-control studies													Quality rating: good (9.5–12), fair (6.5–9), or poor (6–0)
	1. Was the research question or objective in this paper clearly stated and appropriate?	2. Was the study population clearly specified and defined?	3. Did the authors include a sample size justification?	4. Were controls selected or recruited from the same or similar population that gave rise to the cases (including the same timeframe)?	5. Were the definitions, inclusion and exclusion criteria, algorithms, or processes used to identify or select cases and controls valid, reliable, and implemented consistently across all study participants?	6. Were the cases clearly defined and differentiated from controls?	7. If less than 100 percent of eligible cases and/or controls were selected for the study, were the cases and/or controls randomly selected from those eligible?	8. Was there use of concurrent controls?	9. Were the investigators able to confirm that the exposure/risk occurred prior to the development of the condition or event that defined a participant as a case?	10. Were the measures of exposure/risk clearly defined, valid, reliable, and implemented consistently (including the same time period) across all study participants?	11. Were the assessors of exposure/risk blinded to the case or control status of participants?	12. Were key potential confounding variables measured and adjusted statistically in the analyses? If matching was used, did the investigators account for matching during the study analysis?	Total scores: Yes = 1/No = 0.5/NR & NA & CD = 0	
	Yes/No/Not reported (NR) or cannot determine (CD) or not applicable (NA)	Yes/No/Not reported (NR) or cannot determine (CD) or not applicable (NA)	Yes/No/Not reported (NR) or cannot determine (CD) or not applicable (NA)	Yes/No/Not reported (NR) or cannot determine (CD) or not applicable (NA)	Yes/No/Not reported (NR) or cannot determine (CD) or not applicable (NA)	Yes/No/Not reported (NR) or cannot determine (CD) or not applicable (NA)	Yes/No/Not reported (NR) or cannot determine (CD) or not applicable (NA)	Yes/No/Not reported (NR) or cannot determine (CD) or not applicable (NA)	Yes/No/Not reported (NR) or cannot determine (CD) or not applicable (NA)	Yes/No/Not reported (NR) or cannot determine (CD) or not applicable (NA)	Yes/No/Not reported (NR) or cannot determine (CD) or not applicable (NA)	Yes/No/Not reported (NR) or cannot determine (CD) or not applicable (NA)		
Savov et al. 2021 [32]	Yes	Yes	Yes	Yes	Yes	Yes	NR	Yes	NR	Yes	NR	Yes	9	Fair

Change in Hip-Knee-Ankle Angle

**Figure 3:**
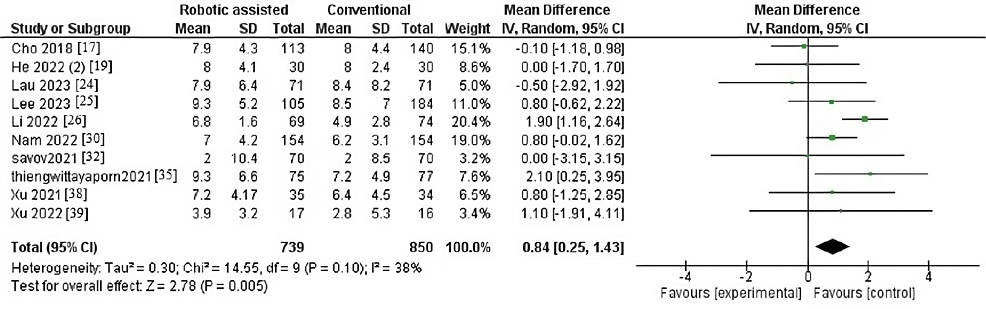
Forest plot of change in hip-knee-ankle angle.

The outcome contained 10 articles with 1,589 patients. The pooled effect estimate showed a significance between robotic-assisted arthroplasty and traditional arthroplasty (MD = 0.84, 95% CI = 0.25-1.43, p = 0.005) (Figure 3). Pooled results were homogeneous (p = 0.10, I^2^ = 38%).

Hospital for Special Surgery

**Figure 4:**
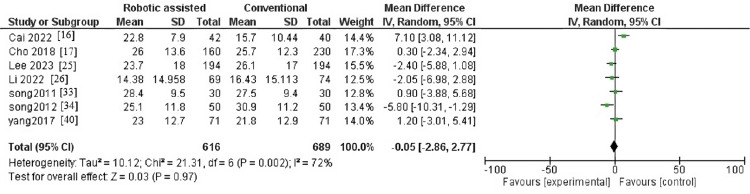
Forest plot of hospital for special surgery.

**Figure 5:**
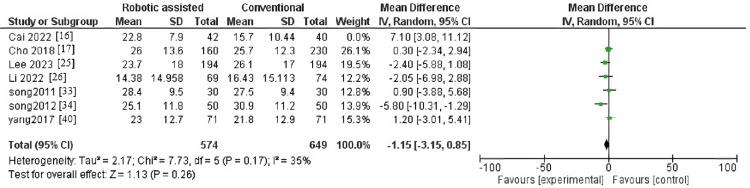
Forest plot of hospital for special surgery after leaving one study out.

The outcome contained seven articles with 1,310 patients. The pooled effect estimate showed no significant difference between robotic-assisted arthroplasty and traditional arthroplasty (MD = -0.05, 95% CI = -2.86-2.77, p = 0.97) (Figure 4). Pooled results were heterogenous (p = 0.002, I^2^ = 72%). The heterogeneity was resolved after the sensitivity analysis by excluding the study by Cai et al. [16], and the result became homogenous (p = 0.17, I^2^ = 35%) (Figure 5).

Change in Oxford Knee Score

**Figure 6:**
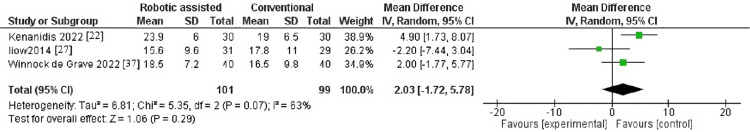
Forest plot of Change in Oxford knee score.

**Figure 7:**
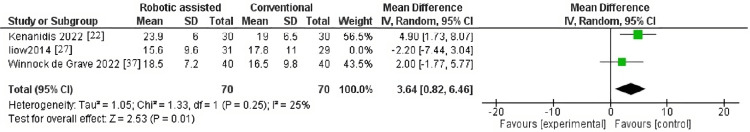
Forest plot of change in Oxford knee score after leaving one study out.

Three articles were included in the outcome with 200 patients. The pooled effect estimate showed a significant difference between robotic-assisted arthroplasty and traditional arthroplasty (MD = 3.64, 95% CI = 0.82-6.46, p = 0.01) (Figure 6). Pooled results were heterogenous (p = 0.07, I^2^ = 63%). The heterogeneity could be resolved after the sensitivity analysis by excluding the study by Liow et al. [27], and the result became homogeneous (p = 0.25, I^2^ = 25%) (Figure 7).

Change in Range of Motion

**Figure 8:**
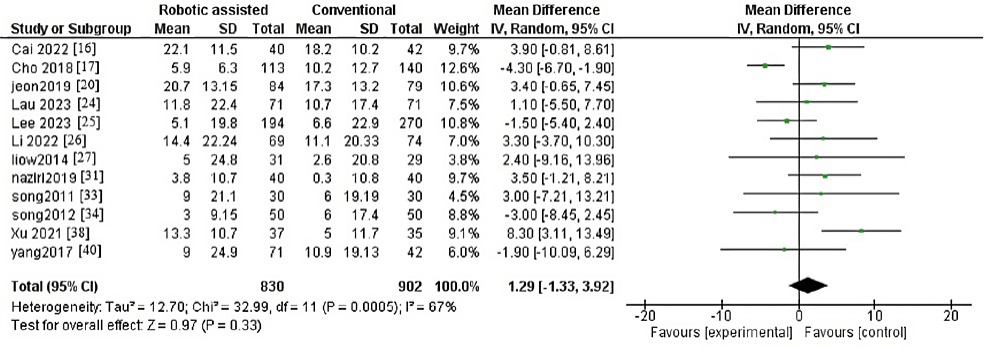
Forest plot of change in range of motion.

The outcome contained 12 articles with 1,732 patients The pooled effect estimate showed no significant difference between robotic-assisted arthroplasty and traditional arthroplasty (MD = 1.29, 95% CI = -1.33-3.92, p = 0.33) (Figure 8). Pooled results were heterogenous (p = 0.0005, I^2^ = 67%).

Operative Time in Minutes

**Figure 9:**
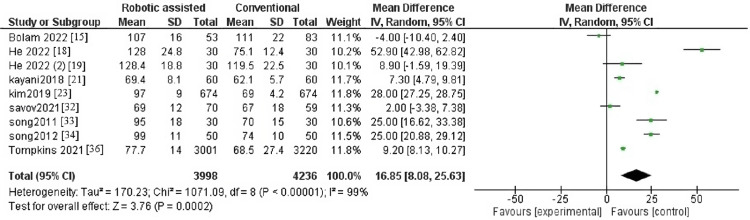
Forest plot of operative time (minutes).

The outcome contained nine articles with 8,234 patients. The pooled effect estimate showed a significant difference between robotic-assisted arthroplasty and conventional arthroplasty (MD = 16.85, 95% CI = 8.08-25.63, p = 0.0002) (Figure 9). Pooled results were heterogeneous (p < 0.001, I^2^ = 99%).

Tourniquet Time

**Figure 10:**
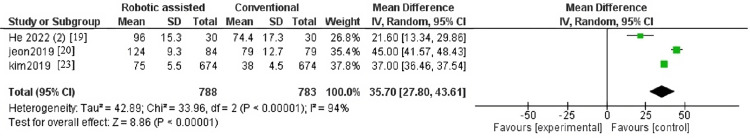
Forest plot of tourniquet time (minutes).

The outcome contained three articles with 1,571 patients. The pooled effect estimate showed a significant difference between robotic-assisted arthroplasty and conventional arthroplasty (MD = 35.70, 95% CI = 27.80-43.61, p < 0.001) (Figure 10). Pooled results were heterogeneous (p < 0.001, I^2^ = 94%).

WOMAC Index

**Figure 11:**
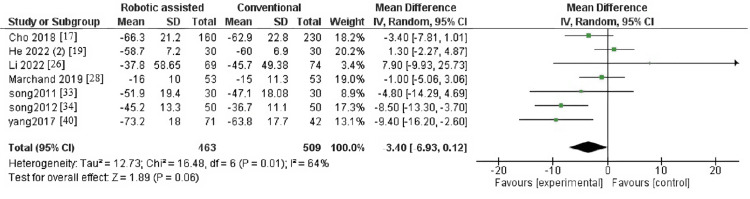
Forest plot of Western Ontario and McMaster Universities Arthritis index.

The outcome contained seven articles with 972 patients. The pooled effect estimate showed no significant difference between robotic-assisted arthroplasty and conventional arthroplasty (MD = -3.40, 95% CI = -6.93-0.12, p = 0.06) (Figure 11). Pooled results were heterogenous (p = 0.01, I^2^ = 64%).

Discussion

Patients typically seek a long-lasting TKA procedure that offers stability, effective pain relief, and enhanced functionality. So far, short-term follow-up studies have not demonstrated any superior clinical outcomes for robotic-assisted TKA when compared to conventional methods. Our comprehensive analysis unveiled several noteworthy findings in the comparative evaluation of conventional TKA and robotic-assisted TKA. Notably, the conventional TKA method exhibited superiority in multiple aspects. First, there was a significant difference in the change of the HKA angle, favoring conventional TKA, indicating that this approach resulted in a more favorable realignment of the lower limb compared to the robotic-assisted method. Second, operation time was notably shorter in conventional TKA, reflecting a more efficient surgical process. Moreover, tourniquet time favored the conventional approach, as robotic-assisted TKA required a longer duration of tourniquet application, temporarily restricting blood flow to the limb. This extended tourniquet time can have potential implications for patient outcomes and recovery. Conversely, when examining various outcome measures, robotic-assisted TKA did not demonstrate significant superiority over the conventional approach. Parameters such as postoperative ROM, the Oxford knee score (a measure of knee function and pain), and the WOMAC index (assessing pain associated with osteoarthritis) did not indicate significant advantages for the robotic-assisted TKA when compared to the conventional method.

The cost of TKA, whether performed conventionally or with the assistance of robotics, can vary significantly depending on several factors, including the location of the medical facility, the surgeon’s fees, the type of implants used, the complexity of the procedure, and whether the patient has insurance coverage. In any possible scenario, the cost of robotic-assisted TKA would be significantly higher than conventional TKA [41-43]. However, Cai et al. [16] reported that the total cost of rehabilitation had no significant difference between the two groups. Furthermore, Lonner et al. [44] found that robotic technology had the potential to play a cost-effective role due to its precision in surgical procedures and the relatively smooth learning curve associated with its use.

Xu et al. [38] noted that in robotic-assisted TKA, a significant portion of the additional surgical duration is allocated to tasks such as setup, femoral and tibial fixation, and alignment. This aspect appears to be a drawback of robotic-assisted TKA that should be improved to reduce the time allocated to non-surgical activities. The primary reasons for prolonged surgery time during the procedure were the intricate process of registering critical bone landmarks and the need for enhancements in the registration success rate. It is worth noting that as surgeons gain proficiency in the procedure, these challenges become less significant, as robotic-assisted TKA is associated with a high range of learning curve [45].

The extended duration of surgery in robotic-assisted TKA holds significant clinical importance, as studies have demonstrated that prolonged surgery times are associated with an elevated risk of peri-prosthetic joint infections [46].

Robotic-assisted TKA significantly reduced pain levels in the two studies by Song et al. [34] and Yang et al. [40]. The overall outcome remains insignificantly in favor of robotic-assisted TKA, with no significant advantage observed. It is worth emphasizing that although robotic-assisted surgery may have the potential to alleviate pain in comparison to conventional methods, individual outcomes can differ. Factors such as the patient’s overall health, the specific surgical procedure, and the surgeon’s expertise are key determinants in assessing the extent of pain relief. Kayani et al. [47] reported that robotic-assisted TKA led to more substantial pain alleviation, enhanced early functional recuperation, and a shorter hospital stay.

Our paper has several notable strengths. We stand as pioneers in conducting a meta-analysis that directly compares conventional and robotic-assisted TKA. One of our significant advantages lies in our extensive sample size, which bolsters the robustness of our findings. Moreover, we meticulously included a wide range of studies, encompassing various types of evidence, to provide a comprehensive overview of the subject. Notably, our study unearthed a major and statistically significant difference between the two approaches, which holds great promise in guiding future orthopedic surgeons and practitioners in their decision-making processes. Our commitment to inclusivity is further underscored by our incorporation of all available RCTs related to this topic, bolstering the strength of our evidence base. To ensure the utmost rigor, we adhered to the guidelines for meta-analyses outlined by Cochrane, enhancing the credibility of our methodology. However, it is important to acknowledge several limitations in our study. High heterogeneity among the included papers posed a challenge, as did variations in study quality. Additionally, discrepancies in follow-up durations, diverse surgical teams, and a multitude of centers spanning different countries introduced significant individual variations. In light of these limitations, we strongly recommend a large-scale RCT directly comparing TKA using both approaches. Such a study should estimate the cost implications and consider long-term outcomes. Aligning follow-up durations among the studies would be essential to provide a more accurate estimation of long-term outcomes. This would facilitate a more comprehensive evaluation of the benefits and drawbacks associated with these surgical techniques.

## Conclusions

Our findings support the superiority of the conventional TKA over the robotic-assisted TKA, as conventional TKA had lower operative time and tourniquet time. Additionally, the HKA angle change was superior in the conventional TKA. The superiority of the robotic-assisted TKA was in the pain outcome taking into consideration that the result was not significant. To provide clearer insights, we recommend a large-scale RCT comparing both TKA methods and assessing costs and long-term outcomes while aligning follow-up durations among studies. This would aid orthopedic decision-making and enhance TKA outcomes.
